# New Operation for Ectropion
*Rust's Magazine. B. 30, H. 3, 1830.


**Published:** 1830-10-01

**Authors:** 


### LVIII.

### NEW OPERATION FOR ECTROPION.*

DR. DIEFFENBACH has invented a new method of operating for this inconvenience and deformity. He makes an incision in the *skin* of the affected eyelid, parallel to the orbit, and occupying as much as two-thirds of the extent of the lid, but in its middle. Having divided the skin and the cellular tissue, these parts are separated from the tarsal cartilage for some extent, and the tarsal conjunctiva is divided, in a direction parallel to the external wound, and for the same length. The tarsal cartilage and the adherent conjunctiva are then laid hold of by forceps introduced through the incision, and their posterior surface having been removed, they are drawn through the wound, and retained between its lips by the hare-lip pin and twisted suture. Three to five points of suture are sufficient, commencing in the centre, cold lotions should be afterwards applied, and the first pins may be withdrawn on the third day, the last on the sixth. Most commonly the wound suppurates a little, but provided that the deep parts unite, the cure is not prevented.

We know not whether the operation is difficult, but we should imagine it to be far from simple under certain circumstances. In the ectropium, for instance, from burn, a very common cause of the deformity, the superficial parts of the palpebra are so puckered and contracted, as to diminish considerably the chances of success from any operation. The loose cellular tissue about the palpebræ is peculiarly prone to suppuration, and the residence of pins in it for five or six days, would be very liable to give rise to so much as to destroy all hopes of a cure. Besides the external wound, the application of pin and suture, and the consequent ultimate contraction of the integuments, seem to us adapted to promote ectropium rather than to remedy it. We leave the point to the experiments of surgeons, if they feel disposed to try any.

* Rust's Magazine. B. 30, H. 3, 1830.

